# Predicting synergistic anticancer drug combination based on low-rank global attention mechanism and bilinear predictor

**DOI:** 10.1093/bioinformatics/btad607

**Published:** 2023-10-09

**Authors:** Yanglan Gan, Xingyu Huang, Wenjing Guo, Cairong Yan, Guobing Zou

**Affiliations:** School of Computer Science and Technology, Donghua University, Shanghai 201600, China; School of Computer Science and Technology, Donghua University, Shanghai 201600, China; School of Computer Science and Technology, Donghua University, Shanghai 201600, China; School of Computer Science and Technology, Donghua University, Shanghai 201600, China; School of Computer Engineering and Science, Shanghai University, Shanghai 200444, China

## Abstract

**Motivation:**

Drug combination therapy has exhibited remarkable therapeutic efficacy and has gradually become a promising clinical treatment strategy of complex diseases such as cancers. As the related databases keep expanding, computational methods based on deep learning model have become powerful tools to predict synergistic drug combinations. However, predicting effective synergistic drug combinations is still a challenge due to the high complexity of drug combinations, the lack of biological interpretability, and the large discrepancy in the response of drug combinations *in vivo* and *in vitro* biological systems.

**Results:**

Here, we propose DGSSynADR, a new **d**eep learning method based on **g**lobal **s**tructured features of drugs and targets for predicting **syn**ergistic **a**nticancer **dr**ug combinations. DGSSynADR constructs a heterogeneous graph by integrating the drug–drug, drug–target, protein–protein interactions and multi-omics data, utilizes a low-rank global attention (LRGA) model to perform global weighted aggregation of graph nodes and learn the global structured features of drugs and targets, and then feeds the embedded features into a bilinear predictor to predict the synergy scores of drug combinations in different cancer cell lines. Specifically, LRGA network brings better model generalization ability, and effectively reduces the complexity of graph computation. The bilinear predictor facilitates the dimension transformation of the features and fuses the feature representation of the two drugs to improve the prediction performance. The loss function Smooth L1 effectively avoids gradient explosion, contributing to better model convergence. To validate the performance of DGSSynADR, we compare it with seven competitive methods. The comparison results demonstrate that DGSSynADR achieves better performance. Meanwhile, the prediction of DGSSynADR is validated by previous findings in case studies. Furthermore, detailed ablation studies indicate that the one-hot coding drug feature, LRGA model and bilinear predictor play a key role in improving the prediction performance.

**Availability and implementation:**

DGSSynADR is implemented in Python using the Pytorch machine-learning library, and it is freely available at https://github.com/DHUDBlab/DGSSynADR.

## 1 Introduction

In complex diseases, monotherapy is likely to encounter with drug-resistance and high cytotoxicity (Güvenç Paltun *et al.* 2021). Through inhibiting multiple disease driving signaling pathways, multi-targeted treatments can greatly enhance efficacy and avoid monotherapy resistance (Chakravarty *et al.* 2022). Therefore, drug combinations have been gradually used to treat various complex diseases. Although drug combination therapy has great clinical value, it still faces many practical challenges.

In early stages, synergistic drug combinations are mainly derived from clinical trials. Such trial-based approach is usually labor-intensive, time-consuming, and risky. With the development of high-throughput screening (HTS) technology, researchers turn to discover novel effective drug combinations by HTS-based methods (Kaemmerer *et al.* 2021). However, the exponential growth of possible drug combinations is an unavoidable roadblock for the HTS-based approach. It is necessary to predict and prioritize a panel of most potent combinations from massive potential combinations for further testing. Machine learning methods provide the opportunity to efficiently explore the large combinatorial space. Existing models such as Random Forest (Breiman 2001), Random Gradient Boosting (Friedman 2001), and Decision Tree (Quinlan 1986) have been widely used for drug combination prediction. The method SyDRa (Li *et al.* 2017) builds a random forest based on three types of features (drug chemical structure, drug–target network and drug-genomics) to predict synergistic anticancer drug combinations. EC-DFR (Lin *et al.* 2022) is an enhanced cascade-based deep forest regression factor method for predicting the synergy scores of drug combinations. These methods are effective for small datasets, whereas it is difficult to achieve good performance on high-dimensional and large-scale datasets. Fortunately, with the publication of large datasets such as NCI-ALMANAC (Sidorov *et al.* 2019) and DrugComb (Zheng *et al.* 2021), deep learning methods have gradually emerged for drug combination prediction. DeepSynergy (Preuer *et al.* 2018) is a pioneer deep learning method for predicting drug combinations, which constructs a DNN based on drug chemical and genomic information. MatchMaker (Kuru *et al.* 2021) takes the chemical features of drugs and the gene expression as the input of a DNN, and utilizes the feature embedding to predict synergy scores of drug combinations. Although these DNN-based methods have made great progress, they usually do not consider complex biological interactions among proteins, drugs, and diseases, which lead to the limitations in explicitly explaining the relevance between different data types and capture the structural information of drugs and targets.

Recently, Graph Neural Network (GNN) (Wang *et al.* 2022a) is exploited to learn feature representation of drug chemical structures and predict drug combinations. DeepDDS adopts Graph Attention Network (GAT) to learn drug features, and integrate the features of cell lines and drug pairs to identify synergistic drug combinations. PRODeepSyn (Wang *et al.* 2022b) utilizes Graph Convolutional Network (GCN) to integrate protein–protein interaction (PPI) networks with genomic data. It combines drug molecular fingerprints and descriptors to predict anticancer synergistic drug combinations. Although these GNN-based methods have achieved superior prediction performance, some limitations still need to be addressed. The GCN model tends to give important features the same weight as other features during propagation, which inhibits the network to extract more critical features. The GAT model tends to limit the attention to partial domains in the graph. The partial feature aggregation is hard to capture global information and more complex node relationships, and also makes the graph computation cost grow significantly as the number of neighbor nodes increases. Therefore, in the feature extraction process, it is necessary to consider the topology structure of different types of data and obtain global structured features with lower complexity.

Here, to tackle these limitations, we propose a new deep learning method DGSSynADR to predict synergistic anticancer drug combinations. To integrate different types of biological data and make the model biologically interpretable, we construct heterogenous graphs based on drug–drug, drug–target, and protein–protein interactions. Meanwhile, using a low-rank global attention (LRGA)-based network, we perform global weighted aggregation of nodes on the heterogeneous graphs to learn global structured features of drugs and targets. For feature extraction of graph structure, the LRGA network effectively reduces the computational complexity of graphs and brings model more generalization ability. Subsequently, instead of using the traditional multi-layer perception (MLP) as a predictor, we connect the learned drug and cell line features, feed them into the bilinear predictor, and predict the synergy score of the drug combination on specific cancer cell lines. On the DrugComb dataset, we compare DGSSynADR with seven competing methods. The extensive results demonstrate that DGSSynADR outperforms the competing methods. Meanwhile, we evaluate the impact of cell line and tissue differences on prediction performance of DGSSynADR. Detailed ablation studies measure the contribution of each module of DGSSynADR. Further case studies validate the synergistic anticancer drug combinations predicted by DGSSynADR, implying that DGSSynADR is an effective drug combination prediction method and could be applied to discover new anticancer synergistic drug combinations.

## 2 Materials and methods

### 2.1 Datasets

#### Drug synergy scores

DrugComb (Zheng *et al.* 2021) is the latest comprehensive drug combination information database, containing the research of NCI-Yearbook, ONEIL and other classic datasets. Both EC-DFR and Matchmaker (Kuru *et al.* 2021) choose the collaborative scores in DrugComb as the source of truth. Similarly, we obtain the drug synergy dataset through DrugComb(v1.5). Compared with DrugComb(v1.4), it adds more drug information covering 751 498 drug combinations and 717 684 single drug screens, corresponding to 21 621 279 independent data points across 2320 cell lines related to 225 cancer types and three infectious diseases. For these drug combinations, DrugComb provides six types of synergy scores, including CSS (Zheng *et al.* 2021), S (Malyutina *et al.* 2019), ZIP (Yadav *et al.* 2015), Bliss (Bliss 1939), Loewe (Loewe 1953), and HSA (Borisy *et al.* 2003). The evaluation models are developed based on different assumptions about the expected effect of noninteraction, which are briefly introduced in the supplementary file. According to the experimental need, we construct a drug–protein relationship network using the data obtained from the drug dataset Chembl and the protein dataset HURI. As the HURI protein dataset does not include all data related to Chembl, we obtain 52 drugs as the intersection of multiple datasets. Then we preprocess the datasets and select 80 877 drug combinations consisting of 52 drugs on 60 cancer cell lines as the benchmark dataset, where each sample comprises two drugs, a cell line and the synergy scores under the six evaluation metrics.

#### Drug features and drug–target interactions (DTI)

The ChEMBL database is a query platform for drug and drug–target data, developed by European Bioinformatics Institute (EBI) (Gaulton *et al.* 2017). The SMILES representation and the therapeutic targets of drugs can be searched by chemical name or index. To better represent chemical structures of drugs, we further utilize the RDKit (Landrum *et al.* 2013) toolkit to process the SMILES of drugs, and calculate the fingerprints, one-hot coding and descriptors for each drug. Specifically, RDKit is open-source toolkit for cheminformatics, which can be used to convert the original SMILEs representation of the drug into coding. Here, it is represented as a 1024D binary vector. Finally, we connect these multiple types of drug representation to obtain the final drug features. The drug–protein graph data are obtained from the DTI relationship data.

#### PPI network

Human Protein Interactome Map (HuRI) is a comprehensive human protein interactome map that contains 53 000 PPIs (Luck *et al.* 2020). ELMo (Heinzinger *et al.* 2019) is a model based on a natural language framework. By modeling protein sequences, it effectively captures the biological features of life language from unlabeled data and forms protein embedding. We utilize the ELMo model to calculate the embedding of protein sequences of the HuRI dataset and generate learnable protein embedding that obey a normal distribution. These two feature representations are subsequently connected as the protein features of the input model.

#### Cell line genomic data

We obtain cell line features such as gene mutation through the Cancer Cell Line Encyclopedia (CCLE) (Ghandi *et al.* 2019) and the LINCS project (Duan *et al.* 2014). Accordingly, we select 1956 genes and input their gene expression profiles to the model.

### 2.2 Framework of the proposed model

**Figure 1.**
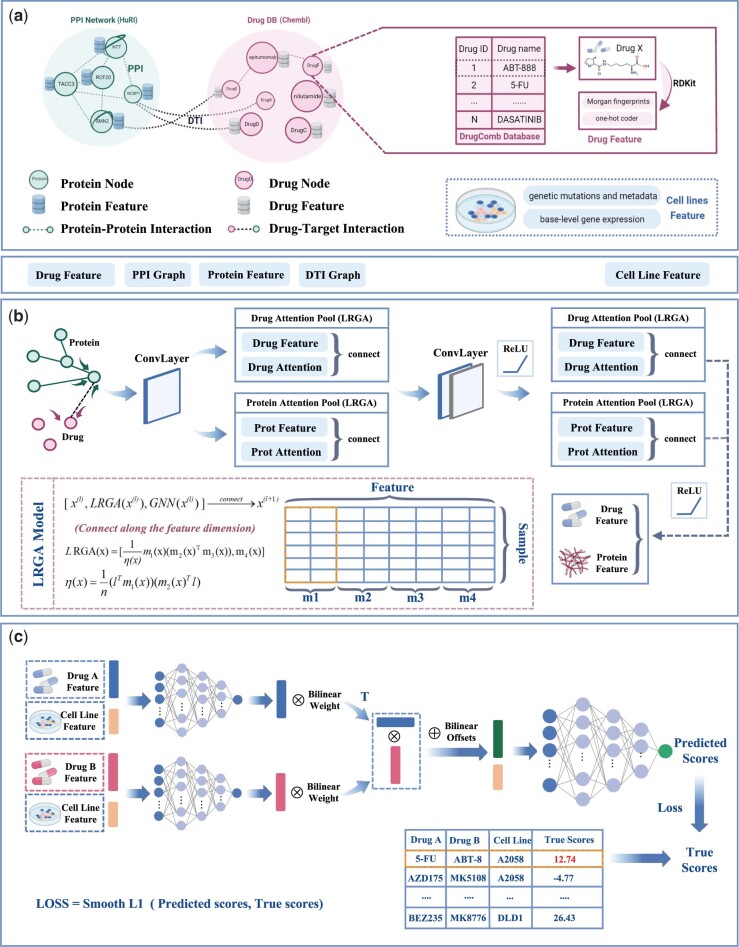
The framework of DGSSynADR. DGSSynADR consists of three major steps, including (a) constructing heterogeneous hectograph of drug–drug, drug–target, and protein–protein; (b) LRGA-based drug and protein feature extraction. LRGA is utilized to perform global weighted aggregation of nodes, and learn feature representations of drugs and proteins from the heterogeneous graph by multi-layer convolutional layers and LRGA-based attention pooling layers; (c) predicting the synergistic scores by bilinear predictors. Two parallel MLPs are trained to learn the feature representations of two drugs on specific cell lines, which are further processed by bilinear operations and jointly fed to the final layer of MLPs. Using Smooth L1 as the loss function, the whole network is trained in an end-to-end way to predict the synergy scores of drug combinations on each specific cancer cell line.

We propose DGSSynADR for predicting anticancer synergistic drug combinations based on the global structural features of drugs and proteins. We model the drug synergy prediction problem as a regression task. For a given drug pair (drug *i*, drug *j*) and cell line *k*, drug *i* and drug *j* are respectively represented by feature vectors $X_{i}$ and $X_{j}$, and cell line *k* is represented by feature vector $C_{k}$. The synergy score of the drug pair obtained from DrugComb is represented as $\mu_{S_{T,\mathrm{ijk}}}$, and the predicted synergy score of the drug pair obtained by DGSSynADR is $\mu_{S_{P,\mathrm{ijk}}}$. Then, the regression problem is to use the feature vectors $X_{i}$, $X_{j}$ and $C_{k}$ to predict the corresponding synergy score $\mu_{S_{P,\mathrm{ijk}}}$. During the training process, the predicted synergy scores gradually approach the target synergy scores. To solve the regression problem, the proposed deep learning method DGSSynADR extracts the global structural features of drugs and proteins, and predicts anticancer synergistic drug combinations. As shown in Fig. 1, DGSSynADR consists of three main steps, including constructing diversified heterogeneous graphs, extracting drug and target features based on LRGA model, and predicting the synergistic scores based on bilinear predictors. Specifically, besides bringing the model better generalization ability, the LRGA network can effectively reduce the complexity of graph computation and feature extraction. The bilinear predictor facilitates the dimension transformation of the features and fuses the feature representation of the two drugs to improve the model performance. DGSSynADR trains the entire network end-to-end using Smooth L1 loss, which is a fast converging and robust loss function.

**Constructing heterogeneous graph for multiple types of biological data.** We respectively construct PPI and DTI networks based on the preprocessed dataset obtained from HuRI and Chembl. Meanwhile, the cell line features such as gene mutation and genomic data are obtained from CCLE and the Cancer Dependency Map (CDM). The synergy scores of each drug pair on specific cell lines are downloaded from the DrugComb database. Based on Loewe scores from DrugComb, drug combinations with Loewe scores larger than 0 are considered as synergistic, otherwise they are regarded as contrastive. We select 52 drugs which are publicly available in both Chembl and DrugComb databases, and set the corresponding protein and drug indexes based on HuRI and Chembl. Using these selected drugs, we construct a heterogeneous network with drug–protein–protein multivariate biological data.

**Figure 2.**
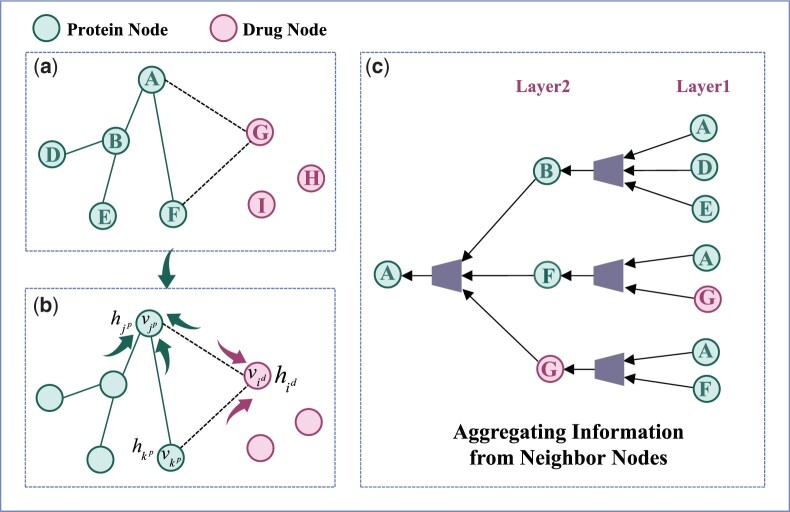
The process of message passing and information aggregation of neighbor nodes.

**LRGA-based graph convolutional network.** As the heterogeneous graph contains multiple types of nonregular data, we adopt a multi-level message passing network (Pei *et al.* 2020) to map different types of information into low-dimensional embedding vector. Message passing networks usually comprises three steps, including aggregating the features of neighboring nodes, updating nodes, and reading out information. In the constructed heterogeneous drug–protein–protein network, we define the node relationship as a set of graphs: G={V,E}, where V denotes the set of vertexes and E denotes the edges. For any node $v_{i}$, the features of this node are represented as $h_{i}^{l-1}$, and the features of edges connecting to neighbor nodes are denoted as $e_{ij}$. The central node updates iteratively along the edges by passing information among neighboring nodes. During this process, the message vector is represented as:


(1) $$m_{i}^{l}=\sum_{j\in N(i)}M_{t}\left(h_{i}^{l-1},h_{j}^{l-1},e_{ij}\right),$$

where N(i) is the neighbor set of the central node $v_{i}$, $M_{t}(\cdot )$ is the message aggregation function (summation operation), and $m_{i}^{l}$ aggregates information from the neighbors of the central node and the edges between nodes. Note that the updating process is a local operation, depending only on the neighbors of node $v_{i}$. As it is unrelated to the size of the graph, the space/time complexity is reduced to O(n), rather than O(E) of the sparse graph (Dwivedi *et al.* 2020). After aggregating messages through the first step, the node updates its hidden features $h_{i}^{l}$ using its features $h_{i}^{l}$ and the message vector $m_{i}^{l}$, and the aggregated information $h_{i}^{l}$ can be defined as:


(2) $$h_{i}^{l}=U_{t}\left(h_{i}^{l-1},m_{i}^{l}\right),$$

where $U_{t}(\cdot )$ is the message update function (summation operation). Specifically, as shown in Fig. 2, the message passing process is different for drug–protein, protein–drug, and protein–protein. As shown in Fig. 2b, the features of drug node $v_{i^{d}}$ are represented as $h_{i^{d}}^{l-1}$. It receives message from its neighbor protein nodes such as $v_{j^{p}}$, and the edge information $e_{i^{d}j^{p}}$ between them. We denote the features of protein node $v_{j^{p}}$ as $h_{j^{p}}^{l-1}$, and the passed messages as $m_{i^{d}j^{p}}^{l}$. Then the messages $M_{d}^{l}$ received by the drug node $v_{i^{d}}$ is defined as:


(3) $$M_{d}^{l}=m_{i^{d}j^{p}}^{l}=\sum_{j\in N(i)}\left(e_{i^{d}j^{p}}⊗f\left(h_{i^{d}}^{l-1},h_{j^{p}}^{l-1}\right)\right),$$

where the function $f\left(h_{i^{d}}^{l-1},h_{j^{p}}^{l-1}\right)$ represents the projection of vector $h_{i^{d}}^{l-1}$ onto $h_{j^{p}}^{l-1}$ direction, which can be understood as a linear mapping relationship.

After the integration of the passed message from the neighbor nodes, the updated feature representation $h_{i^{d}}^{l}$ of drug node *i* can be calculated as:


(4) $$h_{i^{d}}^{l}=M_{d}^{l}+\varepsilon \left(h_{i^{d}}^{l-1}\right).$$

The feature of protein node $v_{j^{p}}$ is represented as $h_{j^{p}}^{l-1}$, which passes messages through the neighbor drug nodes and protein nodes. For the neighbor protein node $v_{k^{p}}$, the feature is denoted as $h_{k^{p}}^{l-1}$. The connected edge between protein $v_{j^{p}}$ and $v_{k^{p}}$ is defined as $e_{j^{p}k^{p}}$, and the passed messages are denoted as $m_{j^{p}k^{p}}$. The messages passing from the neighbor drug nodes to the protein nodes are denoted as $m_{j^{p}i^{d}}^{l}$. The messages received by the protein nodes $v_{j^{p}}$ can be denoted as $M_{p}^{l}$:


(5) $$M_{p}^{l}=m_{j^{p}i^{d}}^{l}+m_{j^{p}k^{p}}^{l}$$

(6) $$M_{p}^{l}=\sum_{i\in N(j)}\left(e_{j^{p}i^{d}}⊗f\left(h_{j^{p}}^{l-1},h_{i^{d}}^{l-1}\right)\right)+\sum_{k\in N(j)}\left(e_{j^{p}k^{p}}⊗f\left(h_{j^{p}}^{l-1},h_{k^{p}}^{l-1}\right)\right).$$

After the protein node $v_{j^{p}}$ receives and integrates the messages from neighbor drug nodes, neighbor protein nodes and the edge information, the feature representation of the protein node can be updated as:


(7) $$h_{j^{p}}^{l}=M_{p}^{l}+\varepsilon \left(h_{j^{p}}^{l-1}\right),$$

where N(⋅) is the set of all neighbor nodes of the central node, *f* denotes a small linear projection, and ε(h) is the self-loop of the node. As the aggregation process aggregates the features of adjacent nodes, we add the self-loop to contain its own feature in the aggregation.

**Figure 3.**
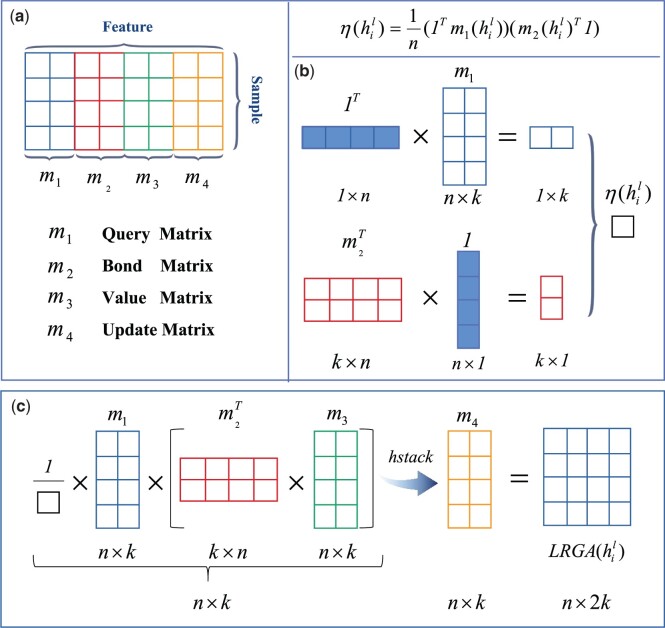
Schematic diagram of matrix operation in LRGA, including (a) $m_{1}$, $m_{2}$, $m_{3}$, and $m_{4}$ are segmented from the feature matrix of the drug, representing Query Matrix, Bond Matrix, Value Matrix, and Update Matrix, respectively; (b) the calculation process of $\eta \left(h_{i}^{l}\right)$; (c) the calculation process of $\mathrm{LRGA}\left(h_{i}^{l}\right)$

After obtaining the initial drug and protein features through the message passing network, we construct a LRGA-based network to extract the global structured features of drugs and proteins. Figure 3 shows the operation process of the matrix in detail. LRGA utilizes the protein/drug feature matrix $h_{i}^{l}$ as input, which can be defined as below:


(8) $$m\left(h_{i}^{l}\right)={\left[m_{1}\left(h_{i}^{l}\right),\;m_{2}\left(h_{i}^{l}\right)\ldots ,m_{n}\left(h_{i}^{l}\right)\right]}^{T}$$

(9) $$\eta \left(h_{i}^{l}\right)=\frac{1}{n}\left(1^{T}m_{1}\left(h_{i}^{l}\right)\right)\left(m_{2}{\left(h_{i}^{l}\right)}^{T}1\right)$$

(10) $$\mathrm{LRGA}\left(h_{i}^{l}\right)=\left[\frac{1}{\eta \left(h_{i}^{l}\right)}m_{1}\left(h_{i}^{l}\right)\left(m_{2}{\left(h_{i}^{l}\right)}^{T}m_{3}\left(h_{i}^{l}\right)\right),m_{4}\left(h_{i}^{l}\right)\right].$$

As shown in Fig. 3, $m_{1}\left(h_{i}^{l}\right)$, $m_{2}\left(h_{i}^{l}\right)$, $m_{3}\left(h_{i}^{l}\right)$, $m_{4}\left(h_{i}^{l}\right):R^{n\times k}$ are the different feature modules split along the feature dimension. *n* is the number of samples, and *k* is the number of features. The $m\left(h_{i}^{l}\right)$ is the general form of $m_{1}\left(h_{i}^{l}\right)$, $m_{2}\left(h_{i}^{l}\right)$, $m_{3}\left(h_{i}^{l}\right)$, and $m_{4}\left(h_{i}^{l}\right)$. Specifically, $m_{1}$ is Query Matrix, $m_{2}$ is Bond Matrix, $m_{3}$ is Value Matrix and $m_{4}$ is Update Matrix. η(⋅) is the normalization factor, and $1={\left(1,1,\ldots ,1\right)}^{T}\in R^{n}$ denotes the transpose of the 1D matrix. Thus, we obtain the feature $h_{i}^{l+1}$ containing information with attention:


(11) $$h_{i}^{l+1}\leftarrow \left[h_{i}^{l},\mathrm{LRGA}\left(h_{i}^{l}\right),\;\mathrm{GNN}\left(h_{i}^{l}\right)\right],$$

where [⋅] denotes connecting along the feature dimension. Here, the attention feature vector obtained by the LRGA model is connected with the previous message passing network.

Compared with the computation process of the attention matrix in the traditional Transformer (Vaswani *et al.* 2017), $m_{1}$, $m_{2}$, and $m_{3}$ in LRGA correspond to the query matrix, key matrix, and value matrix in the self-attention mechanism. Different from the matrix operation in Transformer, LRGA avoids the secondary cost of its obvious computation (Dwivedi *et al.* 2020) and reduces the computation cost to linear by the combination of normalization factor and matrix multiplication. The update matrix module $m_{4}$ in LRGA conforms to the 2-FWL update rule (Lee *et al.* 2019), which can effectively improve the performance of LRGA.

**MLP bilinear-based predictor.** The feature matrix of drugs is defined as $X_{d}\in R^{N_{d}\times F_{d}}$, where $N_{d}$ is the number of drugs and $F_{d}$ is the dimensionality of drug features. The feature matrix is obtained by the feature extraction process through the LRGA-based graph convolution network. The cell line features are merged with the drug features to relieve the dimensional imbalance between the drug and cell line feature vectors. The cell line feature matrix is $X_{c}\in R^{N_{c}\times F_{c}}$, where $N_{c}$ is the number of cell line types and $F_{c}$ is the dimensionality of cell line features.

We define the drug pair and the corresponding cell line as a triple {i,j,k}. Each triple can be split into two corresponding binary groups {i,k} and {j,k}. Let the feature matrices of drug *i* and *j* be $X_{d}^{i}$, $X_{d}^{j}$, and the feature matrix of the corresponding cell line be $X_{c}^{k}$. We connect $X_{d}^{i}$, $X_{d}^{j}$ with $X_{c}^{k}$ along the feature dimension to obtain the binary feature matrix of the drug-cell line. Note that the connection here refers to the connection of the matrix in the vertical direction. The connected feature matrix is subsequently fed into the fully connected MLP to obtain the drug features $X_{d}^{i^{\prime }}$ and $X_{d}^{j^{\prime }}$ in the second stage as follows:


(12) $$X_{d}^{i^{\prime }}=\mathrm{MLP}\left(\left[X_{d}^{i},X_{c}^{k}\right]\right),X_{d}^{j^{\prime }}=\mathrm{MLP}\left(\left[X_{d}^{j},X_{c}^{k}\right]\right).$$

Distinct from the traditional MLP, we adopt a bilinear operation to process the drug features $X_{d}^{i^{\prime }}$ and $X_{d}^{j^{\prime }}$, and feed the combined drug *i* and *j* feature matrix $X_{d}^{i,j}$ into the MLP to predict the synergy score $S_{P,\mathrm{ijk}}$. The process is formulated as follows:


(13) $$X_{d}^{i,j}=\left({\left(w\cdot X_{d}^{i^{\prime }}\right)}^{T}⊗\left(w\cdot X_{d}^{j^{\prime }}\right)⊕b\right)$$

(14) $$S_{P,\mathrm{ijk}}=\mathrm{MLP}\left(\left[X_{d}^{i,j},X_{c}^{k}\right]\right),$$

where *w* is the bilinear weight, *b* is the bilinear operation bias, ⊗ denotes the multiplication operation of the matrix, and ⊕ denotes the addition operation of the matrix. The structural parameters of the MLP are elaborated in the Hyperparameter setting section.

**Loss function.** Although MSE is commonly used loss function between the predicted synergy score and the true synergy score, gradient explosion may occur during the training process as the predicted synergy score differs significantly from the true value at the early stage of the model training. Smooth L1 combines the advantages of L1 and L2 loss (Girshick 2015). It improves the zero-point un-smoothing problem compared with the L1 loss, and is not sensitive to outliers and discrete points compared with L2 loss. Overall, Smooth L1 can avoid the gradient explosion well and is more robust, which is beneficial to better convergence of the model. Therefore, we choose Smooth L1 as the loss function of our model and train the whole network in an end-to-end manner.


(15) $$\mathrm{Loss}=\{\begin{array}{cc} 0.5{\left(S_{P,\mathrm{ijk}}-S_{T,\mathrm{ijk}}\right)}^{2} & if\left|S_{P,\mathrm{ijk}}-S_{T,\mathrm{ijk}}\right|<1\\ \left|S_{P,\mathrm{ijk}}-S_{T,\mathrm{ijk}}\right|-0.5 & \left|S_{P,\mathrm{ijk}}-S_{T,\mathrm{ijk}}\right|\geq 1 \end{array},$$

where $S_{P,\mathrm{ijk}}$ and $S_{T,\mathrm{ijk}}$ respectively denote the predicted and true synergy scores of drug *i* and drug *j* in cell line *k*.

## 3 Results

### 3.1 Hyperparameter setting

The hyperparameters of DGSSynADR include the number of layers and units of each network structure, activation function, and learning rate. As manual parameter screening is infeasible, we adopt the grid search approach to adjust these hyperparameters. To evaluate the performance of different parameter settings, we divide the dataset into the training set, validation set and testing set according to the ratio 6:2:2. We adjust the hyperparameters by the validation set and report the final performance of the model on the testing set. As shown in Supplementary Table S1, we test different values of these hyperparameters via 5-fold cross validation on these benchmark datasets. Specifically, the LRGA network is based on a total of 64 layers, and the model achieves the best performance and stability for different data segments with the bold parameter values.

### 3.2 Performance comparison

To validate the performance of DGSSynADR, we compare it with seven competing methods, including two deep learning-based methods (EC-DFR and MatchMaker) and five classical machine learning methods (XGB, Random Forest, Decision Tree, GBDT, and Bagging Regressor). Specifically, EC-DFR (Lin *et al.* 2022) is an enhanced cascade-based deep forest regression factor method for predicting synergy scores of drug combinations. MatchMaker (Kuru *et al.* 2021) is a deep neural network-based drug synergy prediction algorithm. Among the machine learning methods, Random Forest and Bagging Regressor are classical integrated learning methods based on the Bagging model, and GB, XGB, and GBDT are integrated learning methods based on decision tree, which have shown good performance in synergy prediction of drug combinations. We adopt four widely used metrics to evaluate the predicted synergy scores of drug combinations, including Mean Square Error (MSE), Root Mean Squared Error (RMSE), coefficient of determination ($R^{2}$), and Pearson Correlation Coefficient (PCC). MSE represents the mean squared difference between the predicted scores and true scores, and RMSE is the fitted standard deviation of the regression and is the square root of MSE. $R^{2}$ represents the degree of relevance between the predicted and true scores. The PCC are used to quantify the correlation between the predicted and true scores.

**Figure 4.**
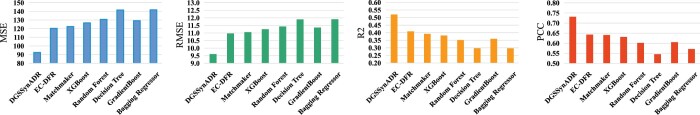
Performance comparison between DGSSynADR and seven competing methods, measured by MSE, RMSE, $R^{2}$, and PCC.

Figure 4 shows the prediction performance of DGSSynADR and seven competing methods under different evaluation metrics. We observe that the proposed DGSSynADR achieves better performance than the competitive methods for all four evaluation metrics. The MSE of DGSSynADR is 92.43, which is 27.87 lower than EC-DFR, 29.99 lower than Matchmaker, and 34.16 lower than XGB. That means DGSSynADR is 23.16% better than EC-DFR and 24.50% better than Matchmaker for the MSE evaluation metric. The same trend is observed when the performance is evaluated using RMSE. The $R^{2}$ of DGSSynADR is 0.5203, which is respectively 11.15%, 12.78%, and 13.90% better than EC-DFR, Matchmaker and XGB. The PCC of our method is 0.7313, which is 8.87% better than that of the suboptimal method EC-DFR, and 9.07% better than Matchmaker. Here, it is noteworthy that the classical machine learning methods, such as XGB, GradientBoost and Random Forest, also obtain competitive performance. Overall, our method achieves superior performance on all four evaluation metrics compared to the two advanced deep learning methods and five classical machine learning methods.

### 3.3 Model performance on different cell lines (tissues)

**Figure 5.**
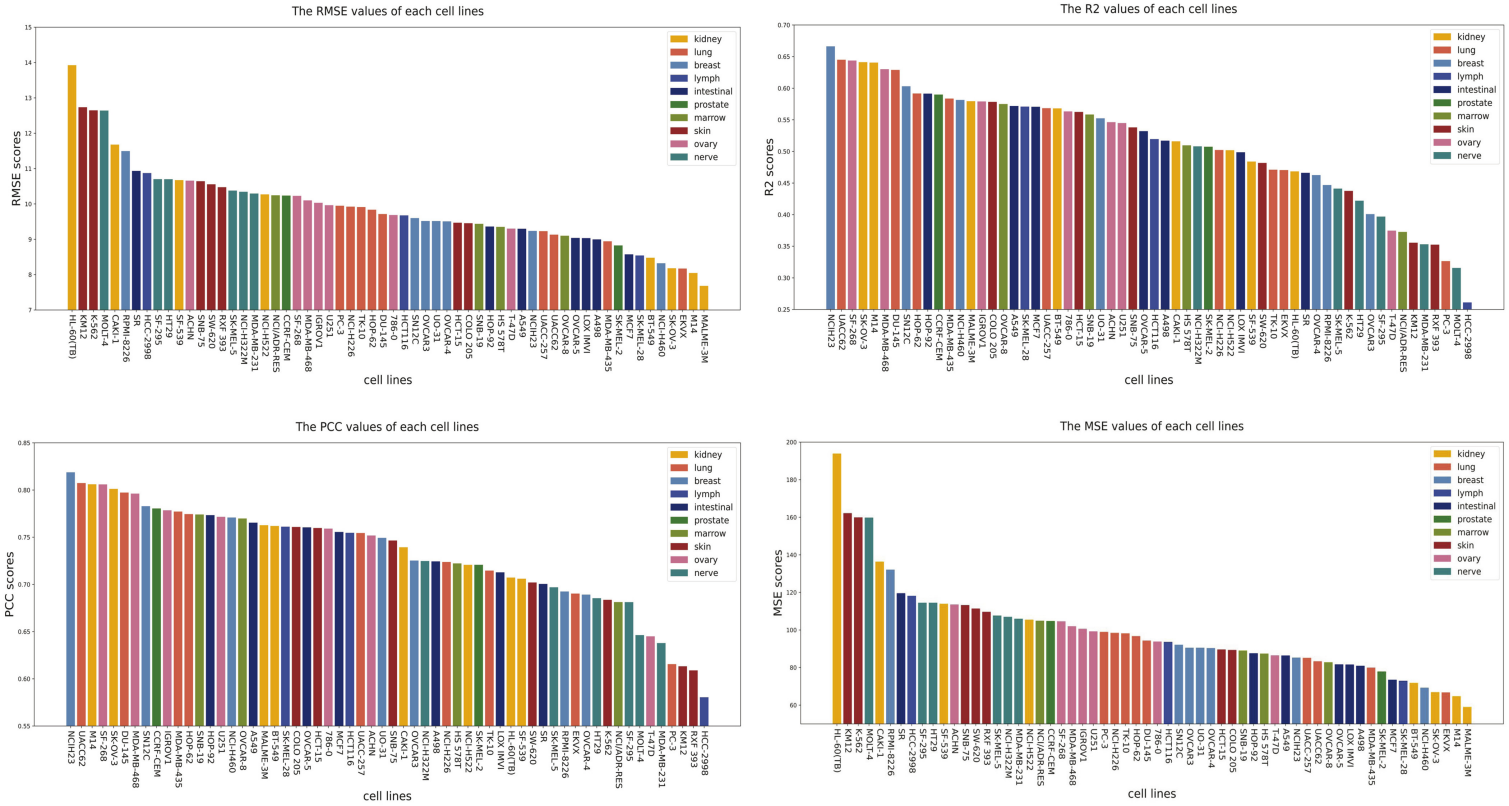
The predicted performance of DGSSynADR on different cell lines.

We further evaluate the predictive performance of DGSSynADR for different cells line separately. Here we choose PCC as the representative evaluation metric. As shown in Fig. 5, the different color bars indicate the tissue types corresponding to that cell line. The PCC of DGSSynADR is higher than 0.75 for 29 of the 60 cell lines. Specifically, it achieves the highest PCC of 0.82 for the cell line *NCIH23*, whereas the cell line *HCC-2998* had the lowest PCC of 0.58. Theoretically, we consider a drug combination with a positive synergy score to be synergistic and the opposite to be antagonistic. The true and predicted synergy scores for *Sunitinib* and *Gefitinib* were −58.88 and −6.94. They were not predicted to be the opposite of the true, but the difference between the predicted and true scores was significant. The problem was also found on cell lines with relatively low PCC, such as *RXF 393* and *PC-3*. Figure 5 shows that DGSSynADR also achieves good performance under the MSE evaluation metric. The top three performance cell lines on MSE were *MALME-3M*, *M14* and *EKVX* (59.03, 64.76, 66.78).

As these cell lines are related to different tissues, we further divide them into 10 tissue subgroups. Subsequently, we evaluate the prediction performance of DGSSynADR on different tissues. Regarding the four evaluation metrics, DGSSynADR achieves better performance on three tissues, including the *ovary*, *breast*, and *lung*. Specifically, the PCC between the predicted scores and the true synergy scores is above 0.70 in all these issues except the intestinal tissue, where the PCC score (0.695) was slightly below 0.70. Overall, DGSSynADR exhibits good performance on most tissues, indicating the capability of DGSSynADR in predicting the synergistic drug combinations for different tissues and cell lines.

### 3.4 Ablation study

DGSSynADR is mainly divided into three modules, including the heterogeneous graphs composed of a variety of biological data, graph attention network based on the LRGA model and bilinear operations. We respectively evaluate their contribution to the prediction performance. Accordingly, seven different variants of DGSSynADR are designed:

- **DGSSynADR-fps** only utilizes the Morgan fingerprint as the original drug feature.
- **DGSSynADR-MSE** uses MSE as the loss function.
- **DGSSynADR-NOPPI** constructs heterogeneous graphs without PPI networks. Accordingly, the drug–protein interaction network (DTI) is also removed with the removal of PPI.
- **DGSSynADR-GNN** adopts basic GNN to learn drug features.
- **DGSSynADR-GAT** uses GAT to learn drug features.
- **DGSSynADR-LRGA-m4** extracts drug features without using GAT to update the matrix structure in LRGA.
- **DGSSynADR-MLP** uses MLP for drug synergy prediction.

Table 1 shows the performance comparison among different variants of DGSSynADR. Compared to DGSSynADR-MSE, DGSSynADR achieves lower MSE and higher PCC (6.60% improvement in PCC), indicating that Smooth L1 loss is more suitable for the model and improves model prediction performance. DGSSynADR achieves a significant (33.71% improvement in PCC) compared to DGSSynADR-fps, which confirms the key role of the one-hot encoding drug feature in the model. The performance of DGSSynADR-NOPPI proves that the lack of PPI network information reduces the prediction accuracy. DGSSynADR-GAT obtains a significant improvement (24.31% in PCC) over DGSSynADR-GNN, indicating that the GAT structure can extract drug features more effectively. Meanwhile, the runtime of DGSSynADR-GAT is about three times longer than DGSSynADR-LRGA. The reason for the difference is that DGSSynADR-LRGA avoids the secondary cost of explicit computation by the attention mechanism. DGSSynADR-MLP performs the prediction task using a simple MLP, and its prediction performance is much poorer than DGSSynADR. This result indicates that the bilinear MLP in DGSSynADR effectively improves the prediction performance. Overall, DGSSynADR achieves the best prediction performance compared to its seven structural variants, and the different variants validate the importance of each component of DGSSynADR on the whole model performance.

**Table 1.** The results of the ablation study.

Method*	MSE	RMSE	$R^{2}$	PCC	Running time (s)
DGSSynADR	**92.44**	**9.61**	**0.52**	**0.73**	3581
DGSSynADR-MSE	117.50	10.84	0.43	0.67	4496
DGSSynADR-fps	170.53	13.06	0.09	0.33	2992
DGSSynADR-NOPPI	115.53	10.75	0.44	0.67	38
DGSSynADR-GNN	159.06	12.61	0.16	0.40	969
DGSSynADR-GAT	122.53	11.07	0.35	0.64	9947
DGSSynADR-LRGA-m4	117.49	10.84	0.41	0.67	41
DGSSynADR-MLP	180.90	13.45	0.13	0.36	**33**

* The highest values are highlighted in bold.

### 3.5 Case study

To verify the reliability of the predictions, we perform a detailed literature search, and present the relevant references for the predicted drug combinations in Table 2. Among these related literatures, we find at least 10 predicted synergistic drug combinations are consistent with clinical or *in vitro* studies. For example, the combination of *Mitoxantrone* and *5-fluorouracil* has validated to be effective in the treatment of advanced breast cancer and is a positive combination option with moderate and manageable toxicity (Samonigg *et al.* 1991). In our study, its predicted synergy score is 4.9814 for the cell line *HS 578T*, associated with breast cancer. The combination of systemic administration of *Temozolomide* and local administration of *Mitozantrone* is reported to reduce the risk of death in adult patients with U87 cells (Glioblastoma) to 50% (Boiardi *et al.* 2008). A synergistic effect of *Erlotinib* and *Quinacrine Hydrochloride* is confirmed in the combination treatment of nonsmall alveolar cell carcinoma (Kulkarni *et al.* 2021), consistent with the predicted results of DGSSynADR. Based on *in vitro* experiments, Gonca *et al.* validated that U87 cells treated with the combination of *5-fluorouracil* and *Ruxolitinib* shows a more prominent decrease in cell viability compared to U87 cells treated with *5-fluorouracil* monotherapy. For this drug combination, the predicted synergy score of DGSSynADR is respectively 7.83 and 68.95 for the cell lines *U251* and *SF-295*, associated with glioblastoma in the dataset. Through clinical studies, Brown *et al.* (Bleiberg *et al.* 1990) demonstrated that *Erlotinib* has significant toxicity in combination with *Temozolomide*, and combining with *Erlotinib* did not show an additional advantage. Consistently, the predicted synergy scores of *Erlotinib* with *Temozolomide* on cell lines *SF-268* and *SNB-75* were −16.78 and −28.38. These results show that the predicted synergy effects of drug combinations by DGSSynADR are consistent with previous findings based on *in vitro* and clinical studies, validating the reliability of the proposed DGSSynADR.

**Table 2.** Case studies of drug combinations in specific cell lines.

Cell line	Drug A	Drug B	Predict scores	True scores	References
HS 578T	Mitoxantrone hydrochloride	5-Fluorouracil	4.98	1.98	Samonigg *et al.* (1991)
SNB-19	Mitoxantrone hydrochloride	Temozolomide	9.79	8.19	Boiardi *et al.* (2008)
HOP-92	Erlotinib HCL	Quinacrine hydrochloride	0.29	6.26	Kulkarni *et al.* (2021)
SF-295	5-Fluorouracil	Ruxolitinib	68.95	2.27	Aksu *et al.* (2020)
HCT-15	Allopurinol	5-Fluorouracil	−21.49	−14.05	Bleiberg *et al.* (1990)
SNB-75	Erlotinib	Temozolomide	−28.38	−33.05	Brown *et al.* (2008)
SNB-75	Ruxolitinib	Temozolomide	28.68	6.53	Qureshy *et al.* (2020)

## 4 Discussion and conclusion

This paper proposes a new deep learning method DGSSynADR for predicting synergistic anticancer drug combinations. DGSSynADR constructs heterogeneous networks based on diverse biological data by combining drug–drug, drug–target, and PPI networks, adopts LRGA model to learn global structured features of drug and protein, and feeds them into a bilinear predictor to predict the synergy scores of drug combinations in specific cancer cell lines. Specifically, LRGA network brings better model generalization ability, and effectively reduces graph computation complexity. The bilinear predictor facilitates the dimension transformation of the features and fuses the feature representation of drug pairs to improve the prediction performance. The loss function Smooth L1 effectively avoids gradient explosion, contributing to better model convergence. To validate the effectiveness of DGSSynADR, we compare it with seven competing methods on the latest large dataset DrugComb. The comparison results demonstrate that DGSSynADR achieves better performance than these methods. To further evaluate the specificity of drug combinations and the performance of DGSSynADR on different cell lines and tissues, we compare MSE, RMSE, $R^{2}$ and PCC on 60 cell lines from 10 tissues. In addition, detailed ablation studies show that the one-hot coding drug feature, the updated matrix m4 of LRGA, and the bilinear operation of the predictor plays a key role in the improvement of the model prediction performance. Finally, case studies indicate that the synergistic drug combinations predicted by DGSSynADR are highly consistent with previous clinical studies. Overall, these extensive experimental results demonstrate that DGSSynADR is an effective drug combination prediction method and could be applied to discover new anticancer synergistic drug combinations.

Although the proposed DGSSynADR method shows promising results in terms of prediction performance and biological interpretability, it is still limited by the experimental data available. Meanwhile, the clinical utility of predicted synergistic drug combination may depend on the individual differences in patients with complex diseases. As more data from clinical trials are added to the drug database, the proposed method may be enhanced to achieve higher accuracy in predicting drug combinations. Furthermore, since previous studies (Kuenzi *et al.* 2020) have investigated the synergistic effects of multiple drugs, we may explore more diverse drug combinations in our future work.

## Supplementary Material

btad607_Supplementary_Data

## References

[btad607-B1] Aksu G , Doğanlar O, Doğanlar ZB. The effect of 5-FU and ruxolitinib on mitochondrial apoptosis in glioblastoma U87 cell line. TMSJ 2020;7:130–9.

[btad607-B2] Bleiberg H , Vanderlinden B, Buyse M et al Randomized phase II study of a combination of cisplatin (DDP), 5-fluorouracil (5-FU), and allopurinol (HPP) versus 5-FU in advanced colorectal carcinoma. An EORTC Gastrointestinal Tract Cancer Cooperative Group Study. Cancer Invest 1990;8:471–5. 2265371 10.3109/07357909009012070

[btad607-B3] Bliss CI. The toxicity of poisons applied jointly. Ann Appl Biol 1939;26:585–615.

[btad607-B4] Boiardi A , Silvani A, Eoli M et al Treatment of recurrent glioblastoma: can local delivery of mitoxantrone improve survival? J Neurooncol 2008;88:105–13. 18283418 10.1007/s11060-008-9540-6

[btad607-B5] Borisy AA , Elliott PJ, Hurst NW et al Systematic discovery of multicomponent therapeutics. Proc Natl Acad Sci USA 2003;100:7977–82. 12799470 10.1073/pnas.1337088100 PMC164698

[btad607-B6] Breiman L. Random forests. Mach Learn 2001;45:5–32.

[btad607-B7] Brown PD , Krishnan S, Sarkaria JN et al; North Central Cancer Treatment Group Study N0177. Phase I/II trial of erlotinib and temozolomide with radiation therapy in the treatment of newly diagnosed glioblastoma multiforme: North Central Cancer Treatment Group Study N0177. J Clin Oncol 2008;26:5603–9. 18955445 10.1200/JCO.2008.18.0612 PMC2651097

[btad607-B8] Chakravarty D , Johnson A, Sklar J et al Somatic genomic testing in patients with metastatic or advanced cancer: Asco provisional clinical opinion. J Clin Oncol 2022;40:1231–58. 35175857 10.1200/JCO.21.02767

[btad607-B9] Duan Q , Flynn C, Niepel M et al Lincs canvas browser: interactive web app to query, browse and interrogate lincs l1000 gene expression signatures. Nucleic Acids Res 2014;42:W449–60. 24906883 10.1093/nar/gku476 PMC4086130

[btad607-B10] Dwivedi VP , Joshi CK, Luu AT et al Benchmarking Graph Neural Networks. *J Mach Learn Res* 2023, 24(43): 1–48.

[btad607-B11] Friedman JH. Greedy function approximation: a gradient boosting machine. Ann Stat 2001;29(5): 1189–232.

[btad607-B12] Gaulton A , Hersey A, Nowotka M et al The chembl database in 2017. Nucleic Acids Res 2017;45:D945–54. 27899562 10.1093/nar/gkw1074 PMC5210557

[btad607-B13] Ghandi M , Huang FW, Jané-Valbuena J et al Next-generation characterization of the cancer cell line encyclopedia. Nature 2019;569:503–8. 31068700 10.1038/s41586-019-1186-3 PMC6697103

[btad607-B14] Girshick R. Fast R-CNN. In: *Proceedings of the IEEE International Conference on Computer Vision*, Santiago: IEEE. 2015, 1440–8.

[btad607-B15] Güvenç Paltun B , Kaski S, Mamitsuka H. Machine learning approaches for drug combination therapies. Brief Bioinform 2021;22:bbab293. 34368832 10.1093/bib/bbab293 PMC8574999

[btad607-B16] Heinzinger M , Elnaggar A, Wang Y et al Modeling aspects of the language of life through transfer-learning protein sequences. BMC Bioinformatics 2019;20:723–17. 31847804 10.1186/s12859-019-3220-8 PMC6918593

[btad607-B17] Kaemmerer E , Loessner D, Avery VM. Addressing the tumour microenvironment in early drug discovery: a strategy to overcome drug resistance and identify novel targets for cancer therapy. Drug Discov Today 2021;26:663–76. 33278601 10.1016/j.drudis.2020.11.030

[btad607-B18] Kuenzi BM , Park J, Fong SH et al Predicting drug response and synergy using a deep learning model of human cancer cells. Cancer Cell 2020;38:672–84.e6. 33096023 10.1016/j.ccell.2020.09.014 PMC7737474

[btad607-B19] Kulkarni NS , Vaidya B, Gupta V. Nano-synergistic combination of erlotinib and quinacrine for non-small cell lung cancer (NSCLC) therapeutics—evaluation in biologically relevant in-vitro models. Mater Sci Eng C Mater Biol Appl 2021;121:111891. 33579503 10.1016/j.msec.2021.111891

[btad607-B20] Kuru HI , Tastan O, Cicek E. Matchmaker: a deep learning framework for drug synergy prediction. IEEE/ACM Trans Comput Biol Bioinf 2021;19(4):2334–44. 10.1109/TCBB.2021.3086702 34086576

[btad607-B21] Landrum G. RDKit: a software suite for cheminformatics, computational chemistry, and predictive modeling. *Greg Landrum*, 2013;8:31.

[btad607-B22] Lee J , Lee Y, Kim J et al Set transformer: a framework for attention-based permutation-invariant neural networks. In: *International Conference on Machine Learning*. Long Beach: PMLR , 2019, 3744–53.

[btad607-B23] Li X , Xu Y, Cui H et al Prediction of synergistic anti-cancer drug combinations based on drug target network and drug induced gene expression profiles. Artif Intell Med 2017;83:35–43. 28583437 10.1016/j.artmed.2017.05.008

[btad607-B24] Lin W , Wu L, Zhang Y et al An enhanced Cascade-based deep Forest model for drug combination prediction. Brief Bioinform 2022;23:bbab562. 35062018 10.1093/bib/bbab562

[btad607-B25] Loewe S. The problem of synergism and antagonism of combined drugs. Arzneimittelforschung 1953;3:285–90. 13081480

[btad607-B26] Luck K , Kim D-K, Lambourne L et al A reference map of the human binary protein interactome. Nature 2020;580:402–8. 32296183 10.1038/s41586-020-2188-x PMC7169983

[btad607-B27] Malyutina A , Majumder MM, Wang W et al Drug combination sensitivity scoring facilitates the discovery of synergistic and efficacious drug combinations in cancer. PLoS Comput Biol 2019;15:e1006752. 31107860 10.1371/journal.pcbi.1006752 PMC6544320

[btad607-B28] Pei H , Wei B, Chang KCC, et al Geom-GCN: Geometric Graph Convolutional Networks. In: International Conference on Learning Representations. 2019.

[btad607-B29] Preuer K , Lewis RP, Hochreiter S et al Deepsynergy: predicting anti-cancer drug synergy with deep learning. Bioinformatics 2018;34:1538–46. 29253077 10.1093/bioinformatics/btx806 PMC5925774

[btad607-B30] Quinlan JR. Induction of decision trees. Mach Learn 1986;1:81–106.

[btad607-B31] Qureshy Z , Johnson DE, Grandis JR. Targeting the JAK/STAT pathway in solid tumors. J Cancer Metastasis Treat 2020;6:27. 33521321 PMC7845926

[btad607-B32] Samonigg H , Stöger H, Kasparek A-K et al Prednimustine combined with mitoxantrone and 5-fluorouracil for first and second-line chemotherapy in advanced breast cancer. Cancer Chemother Pharmacol 1991;27:477–80. 2013118 10.1007/BF00685163

[btad607-B33] Sidorov P , Naulaerts S, Ariey-Bonnet J et al Predicting synergism of cancer drug combinations using nci-almanac data. Front Chem 2019;7:509. 31380352 10.3389/fchem.2019.00509 PMC6646421

[btad607-B34] Vaswani A , Shazeer N, Parmar N et al Attention is all you need. Adv Neural Inf Process Syst 2017;30: 5998–6008.

[btad607-B35] Wang J , Liu X, Shen S et al DeepDDS: deep graph neural network with attention mechanism to predict synergistic drug combinations. Brief Bioinform 2022a;23:bbab390. 34571537 10.1093/bib/bbab390

[btad607-B36] Wang X , Zhu H, Jiang Y et al PRODeepSyn: predicting anticancer synergistic drug combinations by embedding cell lines with protein–protein interaction network. Brief Bioinform 2022b;23:bbab587. 35043159 10.1093/bib/bbab587 PMC8921631

[btad607-B37] Yadav B , Wennerberg K, Aittokallio T et al Searching for drug synergy in complex dose–response landscapes using an interaction potency model. Comput Struct Biotechnol J 2015;13:504–13. 26949479 10.1016/j.csbj.2015.09.001 PMC4759128

[btad607-B38] Zheng S , Aldahdooh J, Shadbahr T et al Drugcomb update: a more comprehensive drug sensitivity data repository and analysis portal. Nucleic Acids Res 2021;49:W174–84. 34060634 10.1093/nar/gkab438 PMC8218202

