# Supplementary material for: Predicting synergistic anticancer drug combination based on low-rank global attention mechanism and bilinear predictor
Source: Bioinformatics. 2023 Oct 9;39(10):btad607. doi: 10.1093/bioinformatics/btad607 (PMC10598574; doi:10.1093/bioinformatics/btad607)
Supplement: btad607_Supplementary_Data [file btad607_supplementary_data.pdf]

Bioinformatics
doi.10.1093/bioinformatics/xxxxxx
Advance Access Publication Date: Day Month Year
Manuscript Category

OXFORD

Genome analysis

Supplementary file

Yanglan Gan [1], Xingyu Huang [1], Wenjing Guo [1], Cairong Yan [1] and Guobing Zou [2,*]

[1] School of Computer Science and Technology, Donghua University, Shanghai, China and
[2] School of Computer Engineering and Science, Shanghai University, Shanghai, China.

Abstract

Hyperparameter setting

The hyperparameters of DGSSynADR include the number of layers and units of each network structure, activation function, learning rate. As manual parameter screening is infeasible, we adopt the grid search approach to adjust these hyperparameters. To evaluate the performance of different parameter settings, we divide the dataset into the training set, validation set and testing set according to the ratio 6:2:2. We adjust the hyperparameters by the validation set and report the final performance of the model on the testing set. As shown in Table 1, we test different values of these hyperparameters via five-fold cross validation on these benchmark datasets. Specifically, the low-rank global attention network is based on a total of 64 layers, and the model achieves the best performance and stability for different data segments with the bold parameter values.

Table 1. Hyperparameter setting

Hyperparameter	Values
batch size	128; 256; 512; **1024**; 2048
learning rate	0.01; 0.001; **0.0001**; 0.00001
dropout rate	0; 0.2; **0.4**; 0.6;
attention rank	32, **64**, 128, 256
embedding layers	16, **32**, 64, 128
predict layers	[1024, 512, 64, 1];**[1024, 64, 32, 1]**; [512, 256, 64, 1]; [512, 256, 32, 1];

Implementation

DGSSynADR is implemented in Python 3 (version 3.6) using PyTorch (version 1.71+cu101). More details of the parameters of the experiment, please refer to the chapter on **Hyperparameter setting**. For every experiment, we run 20 times on datasets and report the average results to ensure data accuracy. All experiments are conducted on NVIDIA Geforce GTX 1080Ti GPU(11G).

The concept of different synergic scores

The combination sensitivity scores (CSS) aim to quantify the efficacy of drug combination therapy in a cross-over design, using the relative IC50 value of the drug combination and its area under the dose-response curve.

S Synergy Score (S) refers to the inhibition rate of more than expected when the two drugs are added at their relative IC50.

The highest single agent (HSA) means that the expected combination effect is equal to the higher single drug effect in the drug combination.

The Loewe additivity model (Loewe) refers to the percentage of the actual inhibitory effect of the two drugs on the basis of the Loewe model that exceeds the expected effect of the relevant theoretical techniques.

Zero Interaction Potency (ZIP) refers to the calculation of the expected effect of a drug combination under the assumption that the two drugs do not enhance each other.

Bliss model(Bliss) refers to the assumption of a random process, in which two drugs act independently, and the expected combination effect can be calculated according to the probability of drug independent events.

Data availability

The datasets are derived from the following sources in the public domain:

The drug synergy scores are obtained from the DrugComb platform `https://drugcomb.org/` . The ChEMBL database from European Bioinformatics Institute query platform `https://www.ebi.ac.uk/chembl/`. The HuRI Protein interaction dataset are obtained from the Center for Cancer Systems Biology `http://www.interactome-atlas.org/`. The cell lines feature datasets are obtained from Cancer Cell Line Encyclopedia platform `https://sites.broadinstitute.org/ccle/` and LINCS project `https://lincs.ed.gov/`.

© The Author 2023. Published by Oxford University Press. All rights reserved. For permissions, please e-mail: journals.permissions@oup.com

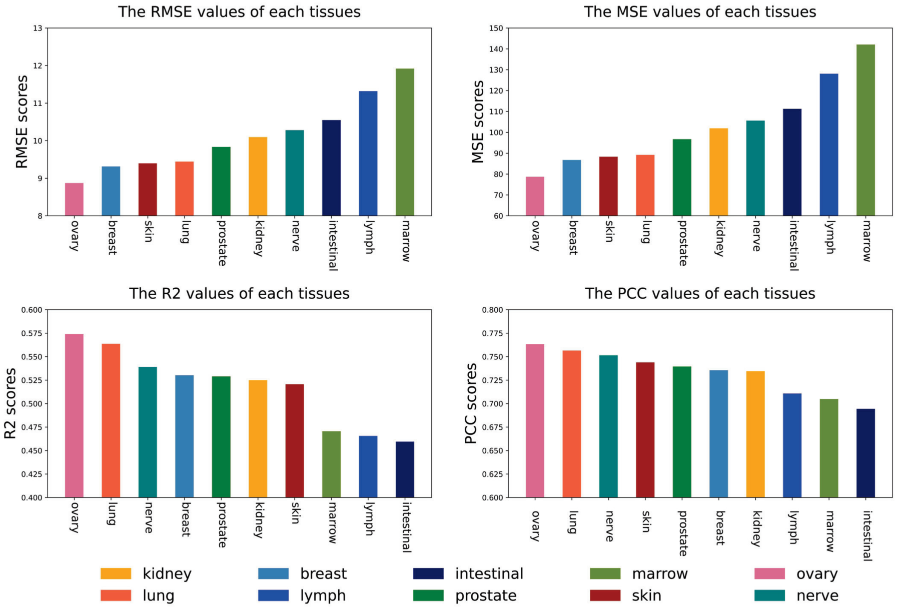

Fig. 1. The predicted performance of DGSSynADR on different tissues

The predicted performance of DGSSynADR on different tissues
